# Contralateral Recurrence and Temporal Trend After First Side Surgery for Primary Spontaneous Pneumothorax: A Multicenter Analysis

**DOI:** 10.3390/jpm16050257

**Published:** 2026-05-09

**Authors:** Antonio Giulio Napolitano, Dania Nachira, Gloria Santoro, Eleonora Coviello, Maria Teresa Congedo, Marco Sanguigni, Domenico Pourmolkara, Chiara Scognamiglio, Leonardo Petracca Ciavarella, Adriana Nocera, Maria Letizia Vita, Felice Mucilli, Jacopo Vannucci, Elisa Meacci, Francesco Puma, Filippo Lococo, Stefano Margaritora

**Affiliations:** 1 Department of Thoracic Surgery, Fondazione Policlinico Universitario “A. Gemelli” IRCCS, Università Cattolica del Sacro Cuore, Largo Agostino Gemelli 8, 00136 Rome, Italy; antoniogiulionapolitano@gmail.com (A.G.N.); dania.nachira@policlinicogemelli.it (D.N.); mariateresa.congedo@policlinicogemelli.it (M.T.C.); kiasco1998@gmail.com (C.S.); leonardo.petraccaciavarella@policlinicogemelli.it (L.P.C.); adriana.nocera91@gmail.com (A.N.); marialetizia.vita@policlinicogemelli.it (M.L.V.); elisa.meacci@policlinicogemelli.it (E.M.); filippo.lococo@policlinicogemelli.it (F.L.); stefano.margaritora@policlinicogemelli.it (S.M.); 2 General Surgery Unit, Fondazione Policlinico Universitario “A. Gemelli” IRCCS, Università Cattolica del Sacro Cuore, Largo Agostino Gemelli 8, 00136 Rome, Italy; gloria.santoro@policlinicogemelli.it; 3 Department of Thoracic Surgery, University of Perugia Medical School, Ospedale Santa Maria della Misericordia, Piazzale Giorgio Menghini 3, 06129 Perugia, Italy; domenico.pourmolkara@aslnapoli1centro.it (D.P.); francesco.puma@unipg.it (F.P.); 4 Department of Anesthesia and Intensive Care, Fondazione Policlinico Universitario “A. Gemelli” IRCCS, Università Cattolica del Sacro Cuore, Largo Agostino Gemelli 8, 00136 Rome, Italy; marco.sanguigni@guest.policlinicogemelli.it; 5 Department of General and Thoracic Surgery, “SS. Annunziata” University Hospital, Via Dei Vestini, 1, 66100 Chieti, Italy; felice.mucilli@unich.it

**Keywords:** primary spontaneous pneumothorax, contralateral recurrence, epidemiology, risk factors

## Abstract

**Background:** Contralateral recurrence following surgically treated primary spontaneous pneumothorax represents clinical concern yet remains poorly understood. This study aims to expand the current understanding by evaluating a large, multicenter cohort over a 10-year period and to determine the true incidence of contralateral recurrence assessing the potential role of clinical factors in risk stratification. **Methods:** A total of 479 patients surgically treated for PSP (2012–2024) across three Italian high-volume centers were retrospectively reviewed. Secondary pneumothorax, patients <18 years old, lung emphysema or intraparenchymal large bullae, and the thoracotomy approach were excluded. The association between categorical variables and contralateral recurrence was assessed using the chi-square (χ^2^) test, while the association with continuous variables was evaluated using the *t*-test. Time to recurrence was analyzed using Kaplan–Meier survival curves. Variables with a *p*-value < 0.05 were considered statistically significant. **Results:** We identified 59 patients who experienced contralateral recurrence: 45 were males, the mean age was 26.66 ± 12.32 and the mean BMI was 22.00 ± 2.92; only 13 were active smokers. Age (*p* < 0.001) and smoking history (*p* = 0.029) were significantly associated with contralateral recurrence in univariate analysis, though these were not confirmed in multivariate analysis. Among the cohort of recurrence, 53 patients only had a recurrence on the contralateral side, with a median time to recurrence of 139 days. The incidence rate ratio (IRR) of recurrence for patients with a mean age of <34 years was 1.23, which translates to a 23% increased risk. No significant impact of age (*p* = 0.25), sex (*p* = 0.67), or smoking (*p* = 0.59) on the time to recurrence on the other side was observed through Kaplan–Meier analysis. The peak incidence for the first episode of PNX surgically treated and contralateral recurrence was observed in October, November and January. **Conclusions:** This study highlights a 12% contralateral recurrence rate after PSP surgery. Younger age is associated with earlier contralateral recurrence. Seasonality may influence recurrence patterns. Further studies should explore underlying mechanisms and preventive strategies.

## 1. Introduction

The incidence of primary spontaneous pneumothorax (PSP) ranges from 7.4 to 18 cases per 100,000 in men and 1.2 to 6 cases per 100,000 in women annually [1].

The true incidence of PSP is unclear; although the majority of initial episodes can be managed conservatively, surgical treatment becomes necessary in cases of recurrence, persistent air leak, bilateral pneumothorax, or high-risk occupations. Indeed, surgery is generally reserved for selected high-risk cases since many will not experience a recurrence.

Even after surgical treatment, ipsilateral recurrence remains a concern, with rates ranging from 2% to 7% depending on the technique and follow-up duration [2]; many factors have been evaluated for recurrence, such as age, smoking, BMI and recent contralateral occurrence [3]. Stopping smoking, including cannabis, is the only proven way to lower recurrence risk and should be strongly encouraged since the first presentation [4,5].

However, contralateral recurrence, defined as the occurrence of PSP on the opposite side to that of the surgical intervention, is comparatively underreported, yet the literature is limited and risk stratification remains poorly defined.

In a previous review on contralateral recurrence, Huang et al. [6] identified low BMI (body mass index) and apical bullae presence on contralateral imaging as potential risk factors. Moreover, a small amount of these included unilateral recurrence, suggesting that the absence of ipsilateral recurrence might paradoxically correlate with higher contralateral risk, due to variables not yet understood.

The pathophysiology behind contralateral recurrence involves bilateral apical bullae disease, as demonstrated in studies [7] using high-resolution computed tomography (HRCT), which documented bilateral multiple or single bullae being an independent risk factor for spontaneous pneumothorax episodes in the contralateral lung.

This study aims to expand the current understanding by evaluating a large, multicenter cohort over a 10-year period. Our objectives were to determine the true incidence of contralateral PSP recurrence, explore timing the and seasonality, and assess the potential role of clinical factors in risk stratification; these patterns might inform individualized follow-up strategies and surgical decision making.

## 2. Materials and Methods

### 2.1. Study Design

The study was approved by the Ethics Committee of the coordinating center (Lazio, Italy, Approval Code: 7824, approved on 4 September 2025) and therefore conducted in accordance with the Ethical standards of the Declarations of Helsinki and its later amendments. In Italy, ethical approval for retrospective observational studies only is generally coordinated at a regional level through designated Ethics Committees. All patients provided informed consent for inclusion in this study.

The Strengthening the Reporting of Observational Studies (STROBE) checklist was followed for reporting the data and results of the present study.

Clinical data of 479 patients who underwent surgical treatment for pneumothorax at 3 Italian high-volume centers, from January 2012 to March 2024, were retrospectively reviewed. For multicenter retrospective studies, after project approval at the coordinating center, participating institutions recognized this and adhered to the same ethical framework and regulations.

The exclusion criteria were secondary pneumothorax, patients under 18 years of age, patients affected by lung emphysema or intraparenchymal large bullae, and the thoracotomy approach.

In all cases, surgery was indicated based on the presence of apical bullae/blebs upon a CT scan, persistent air leaking after chest tube placement, or bilateral pneumothorax.

### 2.2. Surgery and Post-Operative Management

Operations were performed under general anesthesia and single-lung ventilation. Routine blood examination, electrocardiography and anesthesiological evaluation were performed before surgery.

The video-assisted thoracic surgery (VATS) approach was adopted for all cases; the main type of VATS chosen among centers was the uniportal or triportal VATS.

All patients underwent apicectomy and/or atypical resection of the dystrophic area of the lung combined with chemical pleurodesis (with talc aerosolized into the pleural cavity during surgery) or mechanical pleurodesis (performed using scrubbing and electrocautery on the parietal pleura). Only one 28Fr chest tube was placed at the end of surgery, and this was removed usually on third post-operative day if no sign of air leakage was recorded.

### 2.3. Primary and Secondary Outcomes

The primary outcome of the study was to evaluate the incidence of contralateral PSP recurrence in patients who had already undergone surgery on the other side for PSP.

The secondary outcomes investigated potential factors associated with the recurrence of PSP on the contralateral side and evaluated potential factors that correlated with time to recurrence.

### 2.4. Statistical Analysis

For the descriptive analysis of the study sample, preliminary data were characterized. The association between categorical variables and contralateral recurrence was assessed using the chi-square (χ^2^) test, while the association with continuous variables was evaluated using the *t*-test. Univariable regression models were used to evaluate predictors of contralateral recurrence. Only variables with a *p* < 0.2 in the univariable analysis were included in the multivariable regression analysis.

Clinical characteristics regarding the side of the index event and the status of the contralateral lung were assessed postoperatively. Specifically, the variable “Contralateral status” was categorized into “No contralateral” (indicating unilateral involvement at the time of the primary PNP event) and “Contralateral side” (Left or Right), to account for potential synchronous or metachronous findings. These were included as covariates in the multivariable model to evaluate their influence on the long-term risk of recurrence during the follow-up period.

Time to recurrence was analyzed using Kaplan–Meier survival curves. Variables with a *p*-value < 0.05 were considered statistically significant. Statistical analyses were performed using R software (version 4.3.0).

## 3. Results

Out of the 479 patients surgically treated for PSP, 59 patients (12.31%) experienced a recurrence on the contralateral side. Of these 59 patients, 45 (76.27%) were male, with a mean age of 26.66 ± 12.32 and a mean BMI 22.00 ± 2.92; only 13 patients (22.03%) were active smokers. Demographic and clinical data are shown in Table 1.

**Figure 1:**
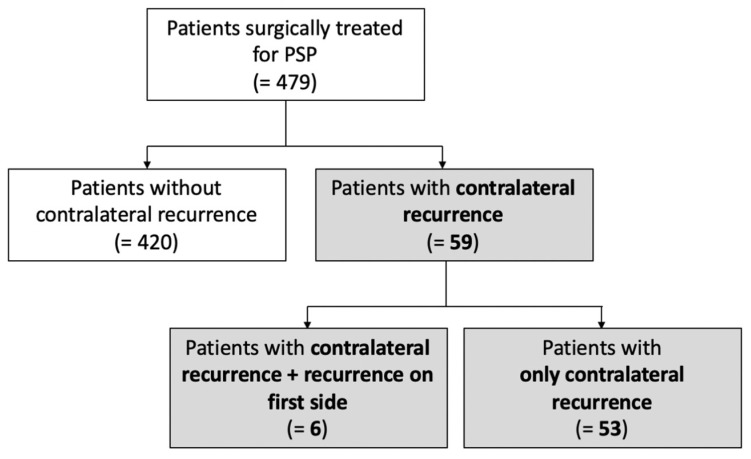
Flowchart describing study population.

Among these 59 patients, 53 patients (11.1%) had only a recurrence on the contralateral side, while 6 (1.3%) had a recurrence on both the ipsilateral side and contralateral side (Figure 1). All patients were clinically monitored for a median follow-up of 140 months.

The analysis showed a numerically lower recurrence-free rate in patients with contralateral disease compared to those without (89.83% vs. 93.57%). However, this difference did not reach statistical significance (IRR (incidence rate ratio): 1.04; 95% CI: 0.95–1.14; *p* > 0.45); see Table 2.

The 53 patients experienced a recurrence only on the contralateral side after a median time of 139 days.

In particular, the IRR of a recurrence for patients with a mean age of <34 years was 1.23 (95% CI: 1.00–1.51), which translates to a 23% increased risk.

Comparing the groups of patients with no recurrences (420 patients) with the 59 patients who had contralateral recurrence, the main factors associated with recurrence upon univariable analysis were as follows: smoking history (*p* = 0.029), age <34 years (*p* < 0.001), contralateral side left (*p* < 0.001). However, these associations were not confirmed in the multivariable model; see Table 3.

**Figure 2:**
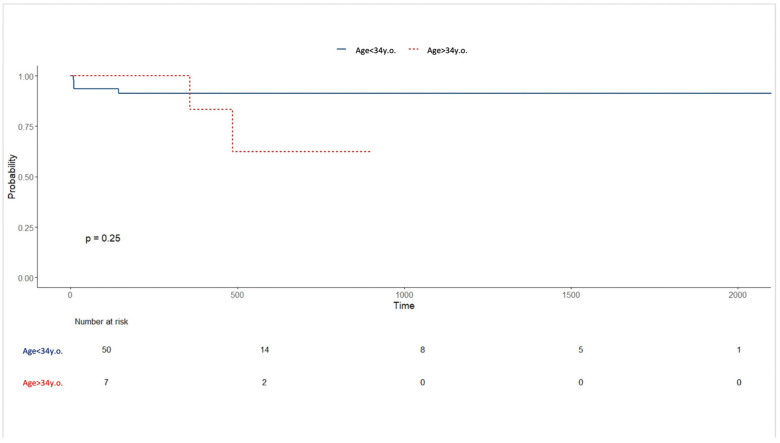
Kaplan–Meier analysis for time to recurrence for age.

**Figure 3:**
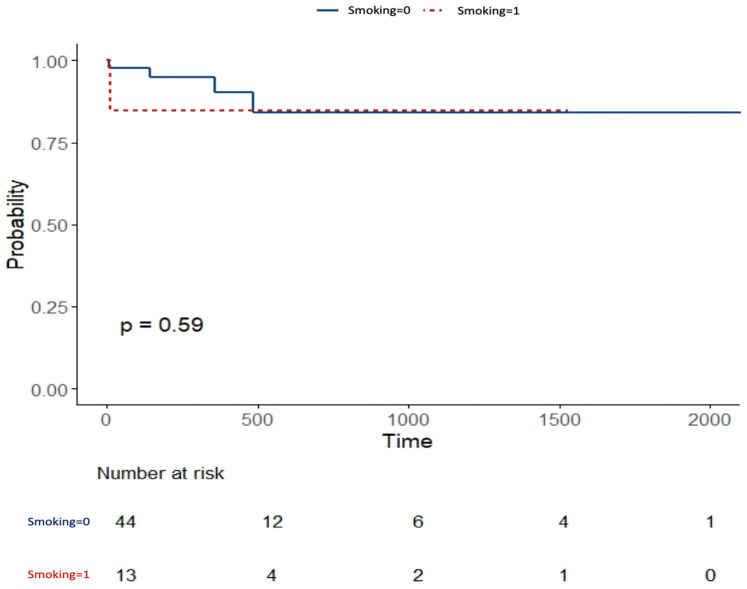
Kaplan–Meier analysis for time to recurrence for smoking.

Kaplan–Meier analysis showed no significant impact of age (dichotomized as ≥34 years and <34 years, according to the median age of the group, *p* = 0.25, Figure 2), sex (*p* = 0.67), or smoking status (*p* = 0.59, Figure 3) on time to recurrence on the other side.

**Figure 4:**
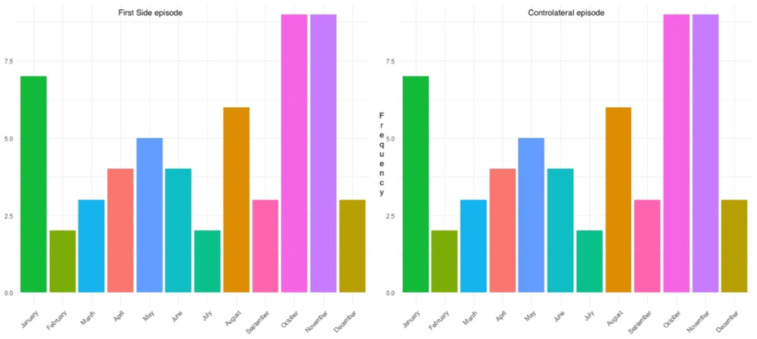
Histograms showing the frequency of the first episode and contralateral episode along the months.

Interestingly, the peak incidences for the first episode of pneumothorax surgically treated and contralateral recurrence were observed in October, November and January, i.e., during the autumn and winter seasons at our latitudes (Figure 4).

## 4. Discussion

The risk of contralateral recurrence in patients with PSP has been historically estimated to range between 5% and 15%, as reported by Lang-Lazdunski et al. and Baumann and Strange [8,9]. However, this baseline risk increases substantially in the presence of radiologically detected contralateral bullae. High-resolution computed tomography (HRCT) has proven valuable in identifying such lesions, and studies have shown that when bilateral blebs are present, the likelihood of a contralateral pneumothorax can increase to as high as 26% [10,11].

This significant increase underscores the prognostic value of preoperative imaging in stratifying recurrence risk and supports the consideration of more aggressive or bilateral approaches in selected high-risk patients. These findings provide a rationale for tailoring surgical strategies based not only on clinical presentation but also on detailed radiological assessment.

Comparison with the previous literature reveals consistent patterns showing a contralateral recurrence rate of 13.3%, similar to our 12.3% [6], highlighting the role of clinical factors such as BMI, smoking status and bilateral bullae (multiple or single) as possible anatomical substrates for recurrence [7]. Notably, most of these events occurred in patients without any prior ipsilateral recurrence, suggesting distinct biological behavior rather than a continuum of disease progression.

In our cohort, although we did not perform systematic preoperative contralateral imaging, the absence of ipsilateral recurrence prior to contralateral events, in nearly 87.68% of patients, aligns with the theory that anatomical predisposition may exist independently on both sides. Based on imaging findings, studies like this suggest [12] that preemptive surgery for asymptomatic contralateral single or multiple bullae can be effective in preventing future contralateral pneumothorax. In selected patients, the bilateral VATS approach may reduce the risk of recurrence on the opposite side, being a safe and effective procedure [13,14].

The seasonality we observed, with nearly 55% of events occurring between October and February, reinforces the effects of external and environmental triggers. An especially interesting finding from our analysis (Figure 4) was that both ipsilateral and contralateral recurrences occurred within exactly the same seasonal timeframes.

Several studies have investigated the role of environmental and seasonal factors in the onset of PSP. Notably, Haga et al. found a significant association between decreased atmospheric pressure and the onset of PSP, suggesting that sudden drops in barometric pressure may act as a trigger [15]; Mishina et al. confirmed a link between weather conditions and PSP occurrence, highlighting atmospheric pressure changes as a contributing factor [16]. Similar findings were reported by Alifano et al. and Bertolaccini et al. [17,18], who observed that changes in atmospheric pressure and temperature may act as external triggers in susceptible individuals. These studies reinforce the theory that environmental fluctuations, particularly barometric pressure changes, can contribute to the pathophysiology of PSP by inducing the rupture of subpleural bullae.

In terms of risk stratification, although younger age and smoking history were associated with recurrence in univariate analysis, neither remained independently significant in multivariate modeling.

However, our data showed that patients under 34 years of age had a markedly higher likelihood of early contralateral recurrence, with a median time of 319 days. This suggests that while traditional factors like age and smoking may not independently predict recurrence, they could still be clinically useful in identifying higher-risk groups.

We employed Kaplan–Meier survival analysis to evaluate time to recurrence considering above and beyond the median time to contralateral recurrence (319 days). Stratifying patients based on this median interval, we analyzed potential risk factors for recurrence occurring before and after this period, including smoking status, sex, age and BMI. However, our analysis did not reveal any statistically significant predictors influencing the timing of contralateral recurrence. This lack of significant findings may be attributed to the limited sample size and the potential influence of external environmental factors, which were not fully accounted for in our study.

Notably, our data indicated a nuanced and controversial relationship between age, smoking status and the timing of contralateral recurrence. Specifically, both smokers and non-smokers exhibited an increased risk of contralateral recurrence within the first 319 days. Interestingly, while the recurrence rate decreased for smokers after this period, it rose sharply with age in non-smokers. This trend may suggest a complex and controversial interplay between intrinsic and extrinsic factors, such as age-related physiological changes, and extrinsic factors, like smoking, in influencing recurrence patterns.

The literature presents mixed findings regarding the role of smoking in PSP recurrence. While smoking is a well-established risk factor for the initial occurrence of PSP [19,20], its impact on contralateral recurrence remains less clear.

Our findings, revealing that younger age is associated with earlier contralateral recurrence of PSP, are in line with the large-scale multicenter study by Yoon et al. [21], which highlighted a disproportionately high incidence of PSP and recurrence in late adolescents and young adults. Specifically, the peak age at first PSP diagnosis in their cohort was between 16 and 19 years, and patients in this age group demonstrated significantly higher recurrence rates (close to 49%) compared with older patients. While their study focused on overall recurrence rather than contralateral events specifically, it nonetheless supports the concept that younger individuals are more prone to early re-pneumothorax episodes, likely due to structural and developmental vulnerabilities such as a low thoracic index or rapid growth phases. This age-dependent susceptibility is concordant with the trend we observed, where the risk of contralateral recurrence increased sharply in younger patients within the first 319 days. Notably, Yoon et al. also emphasize the limited predictive value of smoking in recurrence, which mirrors our findings where the post-319-day decline in recurrence among smokers adds further complexity. Altogether, this comparison reinforces that age is not only an epidemiological determinant of PSP but also a temporal modulator of recurrence dynamics. These temporal and age-related patterns suggest identifiable patients’ subgroups for whom individualized follow-up intensity could be considered. A strength of our study is the multicenter nature and robust follow-up, providing generalizable data across institutions. Limitations include the retrospective design, lack of routine contralateral imaging, potential confounding variables or unmeasured factors not included in the analysis that may better explain the occurrence of contralateral events, and the limited number of patients who experienced recurrence (n = 59). Indeed, the lack of statistical significance of IRR in patients with contralateral recurrence compared with those without (IRR 1.04; 95% CI: 0.95–1.14; *p* > 0.45) may be attributed to the limited number of events recorded for this specific subgroup, which reduced the overall statistical power despite the initially robust cohort size. Furthermore, the present study did not account for external factors, such as meteorological conditions, which might further clarify the relationship between pneumothorax (PNX) and contralateral events. Notably, as illustrated in Figure 4, a higher frequency of cases was observed during the autumn and winter months, suggesting a potential correlation with seasonal atmospheric changes that warrants further investigation. Future prospective studies should incorporate routine HRCT evaluation and consider genetic predisposition markers (e.g., FLCN mutations in Birt–Hogg–Dubé syndrome, Marfan syndrome, vascular type IV Ehlers–Danlos syndrome, alpha-1 antitrypsin deficiency, tuberous sclerosis complex/lymphangioleiomyomatosis, Loeys–Dietz syndrome, cystic fibrosis, homocystinuria, and cutis laxa, among others Marfan, Ehler Danlos), as explored [22,23].

## 5. Conclusions

Contralateral recurrence of PSP occurs in approximately 12% of patients, typically within the first postoperative year.

Younger age patients appear to be associated with an increased risk of earlier contralateral recurrence, suggesting that factors such as age, BMI and smoke attitude may play a role in susceptibility.

The absence of ipsilateral recurrence in the vast majority of patients prior to contralateral events (nearly 90%) suggests that contralateral recurrence may arise independently, rather than as a sequential or progressive pathology. Seasonality may influence recurrence patterns. However, these findings are based on a relatively small number of contralateral events from a multicenter series.

In clinical practice, the key takeaway from our findings is that contralateral recurrence should not be underestimated. Surgeons and pulmonologists should inform patients undergoing PSP surgery of this possibility, particularly younger individuals. A structured follow-up protocol during the first postoperative year, potentially timed around colder seasons, could validate individualized care pathways and optimize recurrence detection and early intervention.

Future prospective studies are warranted to further elucidate underlying mechanisms and to assess the potential role of preemptive or bilateral approaches in select high-risk patients.

## Figures and Tables

**Table 1:** Demographic and clinical data of the cohort.

Variable		Patients (n = 59)
Sex	M	45 (76.27%)
	F	14 (23.72%)
Smoke	Yes	13 (22.03%)
	No	46 (77.96%)
BMI (SD)		22.00 (26.48)
Age (SD)		26.66 (12.00)
Contralateral group	No prior ipsilateral recurrence	53 (89.83%)
	Recurrence after ip-silateral surgery	6 (10.16%)
Contralateral episode side	Right	30 (50.84%)
	Left	29 (49.15%)

**Table 2:** Recurrence-free rates of pneumothorax according to the presence or absence of contralateral recurrence.

Patient Group	Total (n)	Recurrence (n)	Recurrence-Free Rate (%)	95% CI
No Contralateral Disease	420	27	93.57	90.78–95.72
Contralateral Disease	59	6	89.83	79.17–96.18
Total Population	479	33	93.11	90.46–95.21
Measure	**Estimate**	**95% CI**	***p*-value**	
Incidence Rate Ratio (IRR)	1.04	0.95–1.14	0.45 *	
Incidence Odds Ratio (OR)	1.65	0.65–4.18	0.29 *	
Attributable Risk (Exposed)	6.3	−7.20–19.80	-	
Attributable Fraction (%)	7.15	−9.44–21.22	-	

* Indicative *p*-values indicating non-significance results.

**Table 3:** Univariable and multivariable analyses on contralateral occurrence.

Univariable Analysis				
Dependent: Contralateral Episode		No (=420)	Yes (=59)	*p*-Value
Age	Mean (SD)	36.5 (17.8)	26.7 (12.4)	**<0.001**
<34 y.o	**0**	242 (57.6%)	52 (88.1%)	**<0.001**
>34 y.o	**1**	178 (42.4%)	7 (11.9%)	
Sex (F)	**0**	97 (23.1%)	14 (23.7%)	0.871
Sex (M)	**1**	323 (76.9%)	45 (76.3%)	
BMI	**Mean (SD)**	22.2 (3.2)	22.0 (2.9)	0.71
Smoke (No)	**0**	267 (63.6%)	46 (78.0%)	
Smoke (Yes)	**1**	153 (36.4%)	13 (22.0%)	**0.029**
Contralateral side left	**0**	1 (0.2%)	29 (49.2%)	**<0.001**
Contralateral side right	**1**	1 (0.2%)	30 (50.84%)	
Multivariable analysis	**Estimate**	**Std. error**	**z-value**	***p*-value**
Coefficients				
Contralateral side left	−0.68	0.91	−0.74	0.46
No contralateral	−1.04	0.59	−1.75	0.08
Age <34 y.o	0.61	0.38	1.59	0.11
Smoke (Yes)	0.48	0.38	1.27	0.20

## Data Availability

The data cannot be shared publicly to the privacy of individuals that participated in the study. The data presented in this study are available on reasonable request from the corresponding author.

## Abbreviations

The following abbreviations are used in this manuscript:

IRR	Incidence rate ratio
PSP	Primary spontaneous pneumothorax
BMI	Body mass index
HRCT	High-resolution computed tomography
VATS	Video-assisted thoracic surgery
